# Оценка фосфорно-кальциевого обмена и метаболитов витамина D у пациентов с первичным гиперпаратиреозом на фоне болюсной терапии колекальциферолом

**DOI:** 10.14341/probl12851

**Published:** 2021-12-30

**Authors:** И. С. Маганева, Е. А. Пигарова, Н. В. Шульпекова, Л. К. Дзеранова, А. К. Еремкина, А. П. Милютина, А. А. Поваляева, А. Ю. Жуков, В. П. Богданов, Л. Я. Рожинская, Н. Г. Мокрышева

**Affiliations:** Национальный медицинский исследовательский центр эндокринологии; Национальный медицинский исследовательский центр эндокринологии; Национальный медицинский исследовательский центр эндокринологии; Национальный медицинский исследовательский центр эндокринологии; Национальный медицинский исследовательский центр эндокринологии; Национальный медицинский исследовательский центр эндокринологии; Национальный медицинский исследовательский центр эндокринологии; Национальный медицинский исследовательский центр эндокринологии; Национальный медицинский исследовательский центр эндокринологии; Национальный медицинский исследовательский центр эндокринологии; Национальный медицинский исследовательский центр эндокринологии

**Keywords:** первичный гиперпаратиреоз, 1, 25-дигидроксивитамин D, паратиреоидный гормон, дефицит витамина D, колекальциферол

## Abstract

**ОБОСНОВАНИЕ:**

ОБОСНОВАНИЕ. Дефицит (<20 нг/мл) и недостаточность (20–29 нг/мл) витамина D (25-гидроксивитамина D [25(ОН)D]) часто встречаются при первичном гиперпаратиреозе (ПГПТ), но данные о метаболизме витамина D в этой популяции ограничены.

**ЦЕЛЬ:**

ЦЕЛЬ. Изучение метаболитов витамина D и их взаимосвязи с основными показателями фосфорно-кальциевого обмена у пациентов с верифицированным ПГПТ исходно и на фоне однократного перорального приема колекальциферола в болюсной дозе (150 000 МЕ).

**МАТЕРИАЛЫ И МЕТОДЫ:**

МАТЕРИАЛЫ И МЕТОДЫ. Проведено одноцентровое интервенционное динамическое проспективное сравнительное исследование. В исследование вошли 54 человека, распределенных по двум группам: в 1-ю группу включены 27 пациентов с подтвержденным ПГПТ, для сравнения была сформирована 2-я контрольная группа (n=27), сопоставимая по полу (р=0,062). Исследование включало 4 визита, на визите 1 выполнялись лабораторное обследование и прием болюсной дозы колекальциферола, на последующих визитах — динамическое лабораторное обследование.

**РЕЗУЛЬТАТЫ:**

РЕЗУЛЬТАТЫ. Дефицит витамина D (менее 20 нг/мл) определялся у 69% пациентов с ПГПТ. В группе ПГПТ перед приемом колекальциферола наблюдалась прямая ассоциация 1,25(OH)2 D3 с альбумин-скорректированным и ионизированным кальцием, а также между отношением 25(OH)D3 /24,25(OH)2 D3 с паратиреоидным гормоном (ПТГ) и магнием. После приема болюсной дозы колекальциферола на всех визитах в группе ПГПТ уровни 1,25(ОН)2 D3 и соотношение 25(OH)D3 /24,25(OH)2 D3 значимо увеличивались, а 25(OH)D3 /1,25(OH)2 D3 — снижались. При сопоставимой с контролем концентрации 25(OH)D уровни 1,25(OH)2 D3 у пациентов с ПГПТ были исходно на 55% выше, а после приема 150 000 МЕ повышались к 3–7-му дню на дополнительные 23–36%, что на каждом визите было значимо выше таковых значений в группе контроля: 44, 74 и 65% на визите 2, 3 и 4 соответственно (р< 0,05). Использование насыщающих доз в группе ПГПТ не приводило к значимому нарастанию гиперкальциемии и гиперкальциурии, что свидетельствует о безопасности использования данной схемы у пациентов с исходно мягкой гиперкальциемией (альбумин-скорректированный кальций <3 ммоль/л). После приема болюсной дозы колекальциферола ни у одного из участников исследования не фиксировались побочные явления.

**ЗАКЛЮЧЕНИЕ:**

ЗАКЛЮЧЕНИЕ. Впервые проведена комплексная оценка метаболитов витамина D у пациентов с ПГПТ до и после использования болюсной дозы колекальциферола. Полученные результаты свидетельствуют об особенностях метаболизма витамина D при хронической избыточной секреции ПТГ, что безусловно, значимо для понимания патогенеза заболевания, и в дальнейшем эти данные могут быть использованы для разработки терапевтических схем по назначению колекальциферола в указанной популяции.

## Abstract (en)

**BACKGROUND:**

BACKGROUND: Vitamin D (25-hydroxyvitamin D [25(ОН)D]) deficiency (<20 ng/mL) and insufficiency (20–29 ng/mL) are common in primary hyperparathyroidism (PHPT), but data regarding the vitamin D metabolism in this population is limited.

**AIM:**

AIM: The aim of this study is to estimate the vitamin D metabolites and their relationship with the main parameters of phosphorus-calcium metabolism in patients with PHPT at baseline and on the background of a single dose of cholecalciferol 150,000 IU.

**MATERIALS AND METHODS:**

MATERIALS AND METHODS: A single-center interventional, dynamic, prospective, comparative study has been carried out. The study included 54 participants, divided into two groups: the 1st group included 27 patients with confirmed PHPT, the 2nd control group (n = 27), matched on gender (p = 0.062). The study included 4 visits; the baseline laboratory examination and a bolus dose of cholecalciferol were performed at the visit 1, the subsequent visits included a dynamic laboratory examination.

**RESULTS:**

RESULTS: Vitamin D deficiency (<20 ng/ml) was detected in 69% of patients with PHPT. In the PHPT group (before cholecalciferol therapy), there was a direct association of 1.25(OH)2 D3 with albumin-corrected and ionized calcium, as well as between the 25(OH)D3 /24.25(OH)2 D3 ratio with PTH and magnesium. After taking of cholecalciferol, the levels of 1.25(OH)2 D3 and 25(OH)D3 /24.25(OH)2 D3 were significantly increased, and the levels of 25(OH)D3 /1.25(OH)2 D3 were significantly declined at all visits among patients with PHPT. The common 25(OH)D level was comparable to the control group, however the levels of 1,25(OH)2 D3 in patients with PHPT were 55% higher at baseline, and after taking of cholecalciferol 150,000 IU. They remained increased by 3–7 days by an additional 23–36%, significantly higher than those in the control group: 44%, 74% and 65%, at visits 2, 3 and 4, respectively (p<0.05). The taking of 150,000 IU cholecalciferol in the PHPT group did not lead to a significant increase in hypercalcemia and hypercalciuria, which indicates the safety of this dose in patients with mild hypercalcemia (albumin corrected calcium <3 mmol/l). None of the study participants experienced any side effects.

**CONCLUSION:**

CONCLUSION: The completely comprehensive assessment of vitamin D metabolites was carried out for the first time in patients with PHPT before and after using a bolus dose of cholecalciferol. The results confirmed the differences of vitamin D metabolism in chronic excessive secretion of PTH compared to control group, which is new data in the pathogenesis of the disease, and can be used to develop optimal regimens for cholecalciferol taking in this population.

## ОБОСНОВАНИЕ

При первичном гиперпаратиреозе (ПГПТ) чаще, чем в общей популяции, определяется снижение уровня витамина D, распространенность которого в разных исследованиях варьирует в пределах 60–90% [1–3]. В соответствии с действующими российскими клиническими рекомендациями по диагностике и лечению дефицита витамина D под терминами «дефицит» понимается концентрация наиболее распространенного метаболита 25(ОН)D <20 нг/мл (50 нмоль/л), «недостаточность» — от 20 до 30 нг/мл (от 50 до 75 нмоль/л), а «адекватные уровни» — равные или более 30 нг/мл (75 нмоль/л), при которых достигается подавление избыточной секреции ПТГ у большинства индивидуумов. Является ли данная концентрация витамина D целевой для пациентов с ПГПТ, остается достаточно спорным вопросом. В последней версии европейских клинических рекомендаций по ведению пациентов с бессимптомным ПГПТ рекомендуется корректировать дефицит витамина D и поддерживать более низкий оптимальный уровень 25(ОН)D >20 нг/мл (50 нмоль/л), хотя указано, что ряд экспертов предлагают все же целевыми считать значения >30 нг/мл (75 нмоль/л) [4].

25(OH)D — кальцидиол, метаболит витамина D, который чаще всего используется для оценки статуса витамина в организме. Это обусловлено более длительным периодом его полужизни (2–3 нед vs 4 ч у кальцитриола (1,25(ОН)2D3)) и тем, что его концентрация в крови существенно выше, чем таковая 1,25(OH)2D3 (примерно в 1000 раз). Большая часть 25(OH)D прочно связана с витамин D-связывающим белком (DBP), а меньшее количество (10–15%) — с альбумином. Менее 1% циркулирующих метаболитов витамина D существует в свободной, несвязанной форме, но именно эта форма, по сути, является биологически активной [5][6]. При ПГПТ информация по различным формам циркулирующего 25(OH)D (количественные и качественные характеристики) ограничена.

Точные патогенетические механизмы, объясняющие взаимосвязь между ПГПТ и низким уровнем 25(OH)D, остаются не до конца понятными. Известно, что ПТГ стимулирует превращение 25(OH)D в 1,25(ОН)2D3, индуцируя секрецию почечного фермента 1-α-гидроксилазы [7]. Повышенные уровни 1,25(ОН)2D3 при ПГПТ, в свою очередь, могут подавлять дальнейший синтез активного витамина D из предшественников в коже и печени. Период полувыведения 25(OH)D также может быть сокращен при ПГПТ из-за повышенной инактивации кальцидиола в неактивные метаболиты в печени [8]. С другой стороны, сам хронический дефицит витамина D рассматривается в качестве предположительного механизма для запуска гиперплазии околощитовидных желез с последующим приобретением автономной секреции ПТГ и трансформацией в аденому [9]. Имеются данные о том, что тяжесть ПГПТ возрастает при наличии сопутствующего тяжелого дефицита витамина D, так как последний ассоциирован с более высокими показателями ПТГ и кальция, увеличением массы аденомы, более низкой минеральной плотностью костей, повышенным метаболизмом костной ткани и, как следствие, риском низкоэнергетических переломов [10].

## ЦЕЛЬ

Изучение метаболитов витамина D и их взаимосвязи с основными показателями фосфорно-кальциевого обмена у пациентов с верифицированным ПГПТ исходно и на фоне однократного перорального приема колекальциферола в болюсной дозе (150 000 МЕ).

## МАТЕРИАЛЫ И МЕТОДЫ

Место и время проведения исследования

Место проведения. Исследование проведено в ФГБУ «НМИЦ эндокринологии» Минздрава России.

Время исследования. Набор групп и обследование выполнены в период с мая 2019 г. по октябрь 2021 г.

Изучаемые популяции (одна или несколько)

В исследование вошли 54 человека, распределенные по двум группам: в 1-ю включены 27 пациентов с подтвержденным ПГПТ, из них 4 (15%) мужчин и 23 (85%) женщины. Медиана возраста составила 57,5 года [45; 59]. Для сравнения сформирована 2-я контрольная группа (n=27), сопоставимая с 1-й по полу (р=0,062), в которых 10 (27%) мужчин и 17 (63%) женщин. Медиана возраста составила 27 лет [25; 29].

## Критерии включения

Критерии включения в 1-ю группу:

Критерии включения во 2-ю группу:

## Критерии исключения

Критерии исключения из 1-й группы:

Критерии исключения из 2-й группы:

Способ формирования выборки из изучаемой популяции (или нескольких выборок из нескольких изучаемых популяций)

Группа исследования формировалась сплошным методом.

Согласно критериям, в группу контроля был включен 131 человек. После применения критериев исключения с последующим использованием способа псевдорандомизации в настоящее исследование включены 27 человек.

Дизайн исследования

Проведено одноцентровое интервенционное динамическое проспективное сравнительное исследование.

Описание медицинского вмешательства (для интервенционных исследований)

На визите 0 (скрининговый) проводились:

1. сбор анамнеза (на основании медицинской документации и со слов пациента; анализировались данные анкетирования);

2. лабораторное обследование: оценка статуса витамина D;

3. включение в исследование.

На визите 1 (день 0) выполнялись:

1. лабораторное обследование (забор крови из вены, сбор разовой порции утренней мочи);

2. прием лечебной дозы препарата водного раствора колекальциферола (Аквадетрим) 150 000 МЕ перорально.

На визите 2 (день 1), 3 (день 3), 4 (день 7): повторно проводилось лабораторное обследование (забор крови из вены, сбор разовой порции утренней мочи).

Методы

Все лабораторные исследования образцов сыворотки крови и мочи проводились в клинико-диагностической лаборатории ФГБУ «НМИЦ эндокринологии» Минздрава России.

Биохимические параметры сыворотки крови: кальций общий (РИ 2,15–2,55 ммоль/л), альбумин (РИ 34–48 г/л для женщин, 35–50 г/л для мужчин), фосфор (РИ 0,74–1,52 ммоль/л), магний (РИ 0,7–1,05 ммоль/л), креатинин (РИ 50–98 мкмоль/л для женщин, 63–110 мкмоль/л для мужчин) исследованы на автоматическом биохимическом анализаторе ARCHITECТ с8000 (Abbott, CША). Ионизированный кальций определялся расчетным методом (РИ 1,03–1,29 ммоль/л). Исследование интактного ПТГ крови (иПТГ) (РИ 15–65 пг/мл) проводилось на электрохемилюминесцентном анализаторе Cobas 6000 (Roche, Германия), 25(ОН)D (РИ 30–100 нг/мл) — на анализаторе Liaison XL (DiaSorin, Италия). Уровень альбумин-скорректированного кальция рассчитывался по формуле:

общий кальций (ммоль/л) = измеренный уровень кальция сыворотки (ммоль/л) + 0,02 × (40 — измеренный уровень альбумина (г/л)).

Скорость клубочковой фильтрации (СКФ) определялась с учетом возраста и уровня креатинина сыворотки по формуле CKD-EPI 2009. Индекс массы тела (ИМТ) рассчитывался по формуле:

ИМТ = масса тела (кг)/рост (м)2.

Биохимические параметры разовой порции мочи: кальций (РИ 1,7–5,3 ммоль/л), кальций/креатининовое соотношение (ККС) (РИ 0,1–0,8 ммоль/ммоль), фосфор/креатининовое соотношение (ФКС) (РИ 1,4–3,5 ммоль/ммоль) исследованы на автоматическом биохимическом анализаторе ARCHITECТ с8000.

Оценка метаболитов витамина D

Уровни DBP в сыворотке и свободный 25(OH)D измеряли методом иммуноферментного анализа (ИФА) с использованием коммерческих наборов — Assaypro (Миссури, США) и DIAsource (ImmunoAssays S.A., Бельгия) соответственно.

Уровни метаболитов витамина D (25(OH)D3, 1,25(OH)2D3 (РИ 25–66 нг/мл), 3-epi-25(OH)D3 (РИ не разработан) и 24,25(OH)2D3 (РИ 0,5–5,6 нг/мл)) в сыворотке крови определяли методом сверхвысокой производительности жидкостной хроматографии в сочетании с тандемной масс-спектрометрией (UPLC-MS/MS) с использованием собственного разработанного метода, обоснованного на ранее опубликованных другими исследовательскими группами [12–14]. РИ для 25(OH)D3/24,25(OH)2D3 — 7–23 нг/мл, D3 (колекальциферол) — >30 пг/мл.

Образцы сыворотки для измерения DBP (РИ 176–623 мг/л), свободного 25(OH)D и метаболитов витамина D хранились при температуре –80 °C, избегая повторных циклов замораживания-оттаивания.

Для расчета 25(OH)D3/1,25(OH)2D3 (РИ не разработан) и свободного 25(ОН)D (РИ 2,4–35 пг/мл) использовались следующие формулы:

25(OH)D3/1,25(OH)2D3 = 25(ОН)D3 × 1000 / 1,25(OH)2D3.

Свободный 25(ОН)D = (1000 × 25(ОН)D3) / (1 + (9 × альбумин) + (11,2 × DBP)).

Статистический анализ

Статистический анализ проведен в программных пакетах Statistica v.13 (StatSoft, США) и SPSS (IBM, США). Для определения соответствия распределения количественных данных нормальному закону использовался тест Шапиро–Уилка. Описательная статистика количественных показателей представлена медианами, первым и третьим квартилями в виде Me [Q1; Q3]. Сравнение двух независимых групп для количественных данных выполнялось с помощью критерия Манна–Уитни (U-тест), двух зависимых групп — с помощью критерия Вилкоксона. Для сравнения 3 и более зависимых выборок был использован критерий Фридмана. Критический уровень статистической значимости при проверке статистических гипотез принят равным 0,05.

Этическая экспертиза

Исследование (идентификатор ClinicalTrials.gov: NCT04844164) одобрено этическим комитетом ФГБУ «НМИЦ эндокринологии» Минздрава России 10 апреля 2019 г., протокол № 6. Все пациенты подписали информированное согласие на участие в исследовании.

## РЕЗУЛЬТАТЫ

Сравнительный анализ групп до приема колекальциферола

Основные параметры фосфорно-кальциевого обмена в обеих группах на различных визитах представлены в таблице 1.

**Table:** Таблица 1. Параметры фосфорно-кальциевого обмена в исследуемых группах на визитах до и после перорального приема 150 000 МЕ колекальциферола (Me [Q1; Q3])

Визиты	Группа с ПГПТ	Контрольная группа	P, U-тест
Кальций общий, ммоль/л
1	2,71 [ 2,61; 2,80]	2,40 [ 2,34; 2,44]	<0,001
2	2,73 [ 2,60; 2,79]	2,43 [ 2,38; 2,46]	<0,001
3	2,77 [ 2,65; 2,85]	2,42 [ 2,36; 2,49]	<0,001
4	2,74 [ 2,63; 2,84]	2,38 [ 2,34; 2,42]	<0,001
Кальций ионизированный, ммоль/л
1	1,28 [ 1,22; 1,31]	1,10 [ 1,08; 1,12]	<0,001
2	1,27 [ 1,23; 1,3]	1,11 [ 1,09; 1,14]	<0,001
3	1,295 [ 1,24; 1,33]	1,10 [ 1,08; 1,12]	<0,001
4	1,30 [ 1,24; 1,34]	1,11 [ 1,08; 1,12]	<0,001
Альбумин-скорректированный кальций, ммоль/л
1	2,61 [ 2,48; 2,70]	2,29 [ 2,25; 2,33]	<0,001
2	2,60 [ 2,52; 2,71]	2,32 [ 2,29; 2,37]	<0,001
3	2,65 [ 2,55; 2,77]	2,32 [ 2,24; 2,38]	<0,001
4	2,64 [ 2,55; 2,74]	2,29 [ 2,23; 2,34]	<0,001
Фосфор, ммоль/л
1	0,88 [ 0,80; 0,98]	1,15 [ 1,11; 1,27]	<0,001
2	0,89 [ 0,84; 1,01]	1,27 [ 1,18; 1,33]	<0,001
3	0,97 [ 0,85; 1,05]	1,31 [ 1,19; 1,38]	<0,001
4	0,89 [ 0,81; 1,00]	1,28 [ 1,13; 1,36]	<0,001
иПТГ, пг/мл
1	121,40 [ 96,02; 167,60]	31,88 [ 22,21; 38,16]	<0,001
2	115,6 [ 93,4; 156,4]	34,30 [ 26,16; 41,31]	<0,001
3	120,0 [ 91,3; 151,7]	30,55 [ 24,28; 36,40]	<0,001
4	112,80 [ 85,56; 140,90]	29,89 [ 19,92; 42,92]	<0,001
Креатинин, мкмоль/л
1	67,9 [ 64,9; 76,2]	70,6 [ 67,2; 83,3]	0,151
2	69,4 [ 64,8; 75,4]	73,9 [ 66,2; 79,4]	0,071
3	70,3 [ 65,6; 76,2]	70,2 [ 67,8; 78,9]	0,341
4	68,5 [ 62,3; 76,7]	72,0 [ 66,3; 83,3]	0,137
СКФ, мл/мин/1,73 м2
1	89 [ 80; 99]	108 [ 102; 114]	<0,001
2	87 [ 82; 98]	104 [ 98; 114]	<0,001
3	88 [ 78; 94]	107 [ 101; 115]	<0,001
4	91 [ 81; 98]	107 [ 101; 115]	<0,001
Кальций в разовой порции мочи, ммоль/л
1	5,61 [ 3,52; 7,44]	2,32 [ 1,26; 4,29]	<0,001
2	5,89 [ 4,09; 7,05]	3,76 [ 1,99; 5,57]	0,004
3	6,17 [ 3,31; 8,30]	2,60 [ 1,48; 3,79]	0,001
4	5,83 [ 4,35; 8,65]	2,58 [ 1,02; 5,98]	0,001
ККС, ммоль/ммоль
1	0,64 [ 0,37; 1,02]	0,25 [ 0,13; 0,44]	<0,001
2	0,56 [ 0,40; 0,85]	0,37 [ 0,16; 0,60]	0,004
3	0,695 [ 0,505; 0,98]	0,27 [ 0,14; 0,45]	<0,001
4	0,77 [ 0,55; 1,01]	0,20 [ 0,09; 0,51]	<0,001
ФКС, ммоль/ммоль
1	2,66 [ 2,27; 3,21]	1,47 [ 1,24; 2,78]	0,001
2	2,50 [ 2,16; 3,10]	1,83 [ 1,13; 2,33]	0,001
3	2,595 [ 2,29; 3,40]	1,40 [ 1,16; 2,30]	<0,001
4	2,895 [ 2,09; 3,66]	1,60 [ 1,19; 2,42]	<0,001

При сравнении основных лабораторных показателей на визите 1 ожидаемо в группе 1 статистически значимо выше были значения иПТГ, кальция в крови и моче, ККС, ФКС (для всех р<0,001), магния (р=0,016) и ниже — фосфора (р<0,001). Значения СКФ были достоверно ниже в группе ПГПТ (89 мл/мин/1,73 м2 [ 80; 99] vs 108 [ 102; 114], р<0,001).

В исследуемой группе медиана 25(ОН)D составила 17,3 нг/мл [ 13,7; 22,7]. Выраженный дефицит 25(ОН)D (менее 10 нг/мл) был выявлен в 23% случаев (n=6), дефицит 25(ОН)D (от 10 до 20 нг/мл) — в 46% случаев (n=12), недостаточность (>20–30 нг/мл) — в 30,8% (n=8). Уровень 25(ОН)D был достоверно ниже (7,0 нг/мл [ 4,9; 14,2] vs 17,9 нг/мл [ 13,8; 25,8], p=0,044, U-тест), а уровень DBP — достоверно выше (286,5 мг/л [ 276,5; 333,5] vs 234,5 мг/л [ 195; 270], р=0,049) у мужчин по сравнению с женщинами. Различий по остальным метаболитам витамина D среди мужчин и женщин не наблюдалось (p>0,05).

В группе 2 медиана 25(ОН)D составила 20,4 нг/мл [ 15,4; 24,4], выраженный дефицит 25(ОН)D отмечался в 7,4% случаев (n=2), дефицит 25(ОН)D — в 40,8% случаев (n=11), недостаточность — в 51,8% (n=14). Значения 25(ОН)D были сопоставимы у мужчин и женщин (p=0,84), а DBP был достоверно выше у женщин (282 пг/мл [ 246; 391] vs 223 нг/мл [ 197; 259], р=0,035). Различий по остальным метаболитам витамина D среди мужчин и женщин не наблюдалось (p>0,05).

Нами не было выявлено различий по уровню 25(ОН)D (р=0,253) в группах, в том числе при формировании дополнительных подгрупп в зависимости от статуса витамина D (дефицит и недостаточность).

Группы 1 и 2 не отличались по показателям DBP, свободного 25(ОН)D, 25(OH)D3, 3-epi-25(OH)D3, 24,25(OH)2D3 и D3, однако значения 1,25(OH)2D3 и 25(OH)D3/24,25(OH)2D3 были статистически значимо выше в группе 1: 1,25(OH)2D3 — 59,7 нг/мл [ 47,9; 70,7] vs 38,5 нг/мл [ 33,5; 43,3], р<0,001 и 25(OH)D3/24,25(OH)2D3 — 18,02 нг/мл [ 14,2; 30,4] vs 13,1 нг/мл [ 11,0; 15,4], р<0,001). Соотношение 25(OH)D3/1,25(OH)2D3 в группе ПГПТ было значимо ниже (p=0,004).

Сравнительный анализ групп после приема колекальциферола

После приема болюсной дозы колекальциферола ни у одного из участников исследования не фиксировались побочные явления.

Подробная характеристика метаболитов витамина D на различных визитах и их сравнительный анализ между группами представлены в таблице 2. Примечательно, что на всех визитах в группе 1 значения 1,25(ОН)2D3 и 25(OH)D3/24,25(OH)2D3 были статистически значимо выше (р<0,05 для всех), в то время как уровни 25(OH)D3/1,25(OH)2D3 — значимо ниже (р<0,05 для всех), чем у контроля.

**Table:** Таблица 2. Уровни метаболитов витамина D в исследуемых группах на визитах до и после перорального приема 150 000 МЕ колекальциферола (Me [Q1; Q3])

Визиты	Группа с ПГПТ	Контрольная группа	P, U-тест
25(ОН)D, нг/мл
0	17,3 [ 13,7; 22,7]	20,4 [ 15,4; 24,4]	0,253
25(OH)D свободный, пг/мл (не связанный белком)
1	4,74 [ 4,10; 6,16]	6,0 [ 5,0; 6,7]	0,247
2	7,46 [ 5,23; 11,79]	11,7 [ 9,44; 14,03]	0,021
3	10,44 [ 7,34; 14,03]	14,03 [ 10,70; 17,00]	0,009
4	10,33 [ 6,81; 13,14]	12,86 [ 9,91; 16,80]	0,084
DBP, мг/л (белок)
1	257 [ 195; 292]	252 [ 210; 316]	0,487
2	239,5 [ 208; 290]	250 [ 224; 303]	0,532
3	258,5 [ 246; 312]	225 [ 202; 280]	0,013
4	270 [ 250; 319]	263 [ 215; 327]	0,651
25(OH)D3, нг/мл
1	22,4 [ 14,6; 28,8]	21,3 [ 14,8; 23,5]	0,413
2	35,0 [ 23,5; 46,6]	31,0 [ 23,4; 33,8]	0,306
3	40,4 [ 31,8; 44,9]	34,8 [ 31,9; 41,3]	0,420
4	38,4 [ 29,6; 44,5]	37,3 [ 32,1; 42,6]	0,922
3 epi-25(OH)D3, нг/мл
1	0,88 [ 0,6; 1,6]	1,36 [ 1,00; 1,59]	0,068
2	2,45 [ 1,65; 3,25]	2,78 [ 2,50; 3,14]	0,168
3	3,62 [ 2,9; 4,5]	4,37 [ 3,83; 5,67]	0,010
4	3,02 [ 2,5; 3,9]	4,22 [ 3,33; 5,30]	0,001
1,25(OH)2D3, нг/мл
1	59,7 [ 48; 71]	38,5 [ 33,5; 43,3]	<0,001
2	63,75 [ 54,5; 87]	44,3 [ 36,6; 53,6]	<0,001
3	80,4 [ 57; 90]	46,2 [ 41,0; 60,6]	<0,001
4	72,55 [ 55; 91]	43,9 [ 38,1; 52,3]	<0,001
24,25(OH)2D3, нг/мл
1	1,1 [ 0,5; 2,1]	1,63 [ 1,07; 1,93]	0,210
2	1,6 [ 0,6; 2,35]	1,81 [ 1,29; 2,47]	0,223
3	2,1 [ 1,3; 3,0]	2,69 [ 2,12; 3,22]	0,058
4	2,5 [ 1,8; 3,3]	3,07 [ 2,45; 3,57]	0,023
25(OH)D3/24,25(OH)2D3
1	18,0 [ 14,2; 30,4]	13,06 [ 10,99; 15,38]	<0,001
2	24,1 [ 17,5; 42,6]	17,18 [ 14,45; 20,37]	0,003
3	18,7 [ 14,3; 25,4]	13,58 [ 11,46; 14,91]	<0,001
4	15,3 [ 12,6; 19,5]	11,82 [ 10,31; 13,33]	<0,001
25(OH)D3/1,25(OH)2D3
1	367,7 [ 200,0; 451,2]	517,57 [ 372,80; 610,39]	0,004
2	463,0 [ 326,5; 572,5]	675,81 [ 523,72; 788,80]	0,001
3	551,6 [ 380,1; 637,4]	708,96 [ 595,45; 942,92]	0,001
4	572,5 [ 343,5; 680,7]	828,21 [ 658,74; 974,77]	<0,001
D3, пг/мл (колекальциферол)
1	0,01 [ 0,01; 1,98]	0,01 [ 0,01; 2,04]	0,859
2	182,5 [ 148; 198]	226 [ 192; 272]	0,006
3	77,3 [ 69,1; 83,5]	86 [ 57,5; 106]	0,256
4	21,3 [ 17,9; 24,4]	23,5 [ 18,1; 29,7]	0,300

Динамика параметров фосфорно-кальциевого обмена на различных визитах

В группе ПГПТ на фоне насыщения витамином D уровень кальция крови при сравнении с исходным значением максимально повышался на визите 3: для ионизированного и альбумин-скорректированного кальция ∆=0,03 ммоль/л (р=0,008 для ионизированного кальция и р=0,032 для альбумин-скорректированного кальция, рис. 1) (динамика параметров фосфорно-кальциевого обмена на различных визитах представлена в таблице 1).

**Figure:**
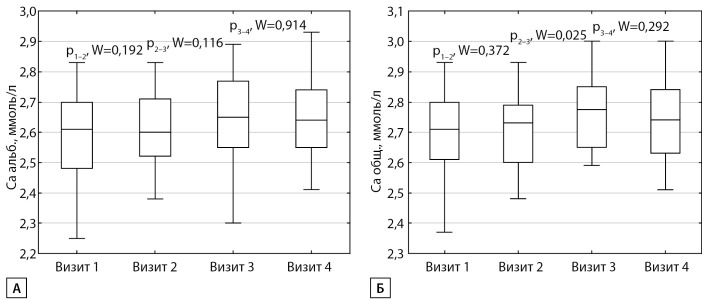
Рисунок 1. Динамика кальциемии в группе первичного гиперпаратиреоза на визитах 1–4: А — альбумин-скорректированный кальций; Б — общий кальций.

Для иПТГ отмечено снижение его уровня на визите 2 у 12 пациентов из группы 1 (∆=-9,93 пг/мл), на визите 4 — у 16 пациентов (∆=-7,06 пг/мл). В 20% случаев на визите 2 было выявлено повышение кальция в моче более 10 ммоль/л со снижением показателей, близким к исходному, на визите 4. Достоверной разницы по уровню кальциурии между визитами выявлено не было (p=0,965, Friedman ANOVA для группы 1 и р=0,973, Friedman ANOVA для группы 2).

Связь метаболитов витамина D с показателями фосфорно-кальциевого обмена на различных визитах

Группа 1.

Исходно уровень 1,25(OH)2D3 напрямую коррелировал с ионизированным кальцием (r=0,47; p=0,02), имел тенденцию к прямой корреляции с альбумин-скорректированным кальцием (r=0,39, p=0,05), а соотношение 25(OH)D3/24,25(OH)2D3 — прямую связь с уровнем иПТГ, а также между 25(OH)D3/24,25(OH)2D3 и иПТГ (r=0,60; p=0,001) и магнием (r=0,60; p=0,001). Значения иПТГ и магния были достоверно выше при более низких концентрациях свободного 25(OH)D (r=-0,67, p=0,002 для иПТГ и r=-0,523, p=0,026 для магния), 3-epi-25(OH)D3 (r=-0,46; р=0,02), 24,25(OH)2D3 (r=-0,58; р=0,02) и отношения 25(OH)D3/1,25(OH)2D3 (r=-0,71, р<0,001 для иПТГ и r=-0,587, p=0,002 для магния). Для СКФ только на 1 визите прослеживались ассоциации с некоторыми метаболитами витамина D: выявлена прямая связь с 25(OH)D3/24,25(OH)2D3 (r=0,47, р=0,02) и обратная связь с 24,25(OH)2D3 (r=-0,461, р=0,018).

На 2 визите ассоциации между 1,25(ОН)2D3 с уровнем альбумин-скорректированного кальция получено не было. Более низкие показатели 3-epi-25(OH)D3 ассоциировались с гиперкальциемией на следующий день после приема болюсной дозы колекальциферола (r=-0,47, p=0,02 для альбумин-скорректированного кальция), а 25(OH)D3/1,25(OH)2D3 — с более высоким иПТГ (r=-0,42, p=0,04).

На 3 и 4 визитах 1,25(OH)2D3 напрямую коррелировал с уровнем иПТГ (r=0,59; p=0,002 и r=0,58; p=0,002 соответственно), так же, как и 25(OH)D3/24,25(OH)2D3 (r=0,44; p=0;026 и r=0,55; p=0,004 соответственно). Обратная корреляция на визитах 3 и 4 наблюдалась с иПТГ у 25(OH)D3/1,25(OH)2D3 (r=-0,57; p=0,003 и r=-0,55; p=0,004).

На визитах 3 и 4 уровни ионизированного кальция обратно коррелировали с 24,25(OH)2D3 (r=-0,55; р=0,004 и r=-0,47; р=0,02 соответственно) и 25(OH)D3/1,25(OH)2D3 (r=-0,48; р=0,02 и r=-0,40; р=0,04). На визите 4 выявлена прямая взаимосвязь между ионизированным кальцием и свободным 25(OH)D (r=-0,50; р=0,03). Фосфор сыворотки крови обратно коррелировал с 25(OH)D3/24,25(OH)2D3 (r=-0,39; р=0,047) на визите 1, и выявлена его прямая зависимость с D3 на визите 3 (r=0,49; p=0,01). Связи метаболитов витамина D с кальциурией на всех визитах не выявлено.

Группа 2.

На визите 1, так же как и в группе ПГПТ, выявлена прямая зависимость между 1,25(OH)2D3 и альбумин-скорректированным кальцием (r=0,40; p=0,04), а также между отношением 25(OH)D3/24,25(OH)2D3 и иПТГ (r=0,67, р<0,001). Аналогично в данной группе выявлена обратная связь иПТГ с 3-epi-25(OH)D3 (r=-0,61; р=0,001), со свободным 25(OH)D (r=-0,50; р=0,007), с 24,25(OH)2D3 (r=-0,70; р<0,001), с отношением 25(OH)D3/1,25(OH)2D3 (r=-0,56; р=0,002). На визите 3 сохранялась обратная взаимосвязь иПТГ со свободным 25(OH)D (r=-0,40; р=0,04), с 24,25(OH)2D3 (r=-0,44; р=0,02) и отношением 25(OH)D3/1,25(OH)2D3 (r=-0,47; р=0,01).

В отличие от группы ПГПТ в контрольной группе на визите 1 была выявлена прямая зависимость между D3 и кальцием: для ионизированного кальция r=0,41; р=0,04, для альбумин-скорректированного кальция r=0,46; р=0,02. Данная тенденция сохранилась только для ионизированного кальция на визите 2 (r=0,45; р=0,02). Также на визите 2 прямая зависимость была выявлена между фосфором со следующими показателями: 3-epi-25(OH)D3 (r=0,40; р=0,04), 24,25(OH)2D3 (r=0,53; р=0,005), отношением 25(OH)D3/1,25(OH)2D3 (r=0,46; p=0,02) и D3 (r=0,45; р=0,02).

На визите 4 отмечалась обратная взаимосвязь кальция с 3-epi-25(OH)D3: для ионизированного кальция r=-0,40; р=0,04, альбумин-скорректированного кальция r=-0,39; р=0,04), а также альбумин-скорректированного кальция с 24,25(OH)2D3 (r=-0,47; р=0,01) и ионизированного кальция с отношением 25(OH)D3/1,25(OH)2D3 (r=-0,42; р=0,03). Значения альбумин-скорректированного кальция напрямую коррелировали с отношением 25(OH)D3/24,25(OH)2D3 (r=0,40; р=0,04). Прямая взаимосвязь с СКФ была отмечена у некоторых метаболитов витамина D на визите 4: для свободного 25(OH)D (r=0,43; р=0,02), 24,25(OH)2D3 (r=0,46; р=0,01) и отношения 25(OH)D3/1,25(OH)2D3 (r=0,382; р=0,050).

Корреляция кальциурии с метаболитами витамина D была выявлена на визитах 2 и 4: после приема болюсной дозы колекальциферола на визите 2 отмечена обратная зависимость между концентрацией кальция в моче и 24,25(OH)2D3 (r=-0,40; р=0,04), а также положительная с соотношением 25(OH)D3/24,25(OH)2D3 (r=0,44; p=0,02); на визите 4 — прямая связь между кальциурией и D3 (r=0,49, р=0,01). Отрицательная связь прослеживалась между 1,25(OH)2D3 и ФКС на визите 1 (r=-0,57; p=0,002) и на визите 2 (r=-0,53; р=0,004). Также на визите 2 отмечена обратная корреляция между 25(OH)D3/1,25(OH)2D3 и ККС (r=-0,41; р=0,03), а прямая зависимость — между 25(OH)D3/24,25(OH)2D3 и ККС (r=0,44; р=0,02) и между D3 и ККС (r=0,42; р=0,04), которая сохранялась и на визите 4 (r=0,63; р=0,001).

## ОБСУЖДЕНИЕ

Репрезентативность выборок

Выборка пациентов с ПГПТ в исследовании соответствует целевой популяции пациентов с этим заболеванием, что характеризуется основными показателями и их взаимосвязями. Контрольная группа сформирована сопоставимой по полу. Более молодой возраст индивидуумов группы контроля позволил нивелировать возможные возраст-ассоциированные факторы, связанные в том числе с бременем неучтенной полипрагмазии и различных хронических заболеваний. Каких-либо специфических факторов (социальных, экономических, культурных, др.), способных повлиять на внешнюю валидность выводов исследования, не отмечено.

Сопоставление с другими публикациями

ПТГ — один из основных регуляторов как фосфорно-кальциевого обмена, так и метаболизма витамина D. Многие данные свидетельствуют о том, что дефицит витамина D при ПГПТ является фактором, стимулирующим увеличение секреции ПТГ [15] и большую активность заболевания [16]. В последнее десятилетие значимо увеличилось выявление бессимптомных форм и нормокальциемических вариантов ПГПТ, что связано c повышением доступности лабораторного определения кальция и ПТГ. Поскольку пациенты с ПГПТ могут в этих случаях наблюдаться годами без хирургического вмешательства, последствия дефицита витамина D имеют особое значение [17, 18].

В нашем исследовании у 69% пациентов с ПГПТ уровень 25(ОН)D соответствовал дефициту витамина D (менее 20 нг/мл) и был достоверно ниже среди мужчин, что согласуется с данными зарубежных исследований [1–3]. Отсутствие статистических различий в исходных уровнях витамина у пациентов с ПГПТ и контроля, определенных как скрининговым иммунохемилюминесцентным методом, так и LC-MS/MS, может происходить от характерной для популяции высокой распространенности дефицита и недостаточности витамина D в Российской Федерации и является фактором преимущества для дальнейшей сравнительной оценки динамики уровней самого витамина D и его метаболитов до и после приема нагрузочной дозы колекальциферола [19]. Выдвигается гипотеза, что уровень 25(ОН)D в крови при ПГПТ не является «истинным» дефицитом витамина D, а может быть следствием ускоренной метаболической конверсии 25(ОН)D [20, 21], большей деградацией до 24,25(OH)2D и/или снижением циркулирующих уровней DBP [22]. По данным U.M. Kabadi и соавт., у лиц с ПГПТ уровень 1,25(OH)2D3 был выше на 39–147%, чем у лиц с нормальным и низким уровнем 25(ОН)D. При этом 25(ОН)D у пациентов с ПГПТ был снижен в среднем до 18±3 нг/мл, но существенно не отличался от группы с дефицитом витамина D без ПГПТ (средний уровень 15±2 нг/мл) [23]. В настоящем исследовании мы получили, что при одинаковом 25(OH)D уровни 1,25(OH)2D3 у пациентов с ПГПТ были исходно на 55% выше, а после приема 150 000 МЕ они повышались к 3–7-му дню на дополнительные 23–36%, что на каждом визите было значимо выше таковых значений в группе контроля: 44, 74 и 65% на визите 2, 3 и 4 соответственно (р<0,05). Эти данные подтверждают наличие повышенной ПТГ-ассоциированной конверсии 25(OH)D в 1,25(OH)2D3.

В исследовании L. Meng и соавт. подтверждены более низкие уровни общего 25(OH)D при ПГПТ, что ассоциировано с более низкими концентрациями DBP (р<0,001). Свободный 25(OH)D и 1,25(OH)2D3 не отличались, а уровни свободного 1,25(OH)2D3 были примерно на 26% выше у пациентов с ПГПТ по сравнению с контролем (р<0,001). Пациенты с ПГПТ продемонстрировали значительно более высокий коэффициент активации витамина D, рассчитанный как соотношение 1,25(OH)2D/25(OH)D (р<0,01), хотя общее количество 25(OH)D было ниже, чем в контроле [24]. Свободный 1,25(OH)2D3 отрицательно коррелировал с уровнями DBP (p<0,01) и вместе с коэффициентом активации положительно коррелировал с уровнями ПТГ и кальция крови (р<0,01). Данные нашего исследования не позволяют подтвердить тот факт, что при ПГПТ имеют место более низкие концентрации DBP, так как не выявлено их отличий между группами. Выявленная гендерная разница в концентрации данного белка была очевидна в обеих группах нашего исследования и могла стать причиной выявленных отклонений в исследовании L. Meng и соавт. Соответственно, маловероятно, что изменение уровней DBP при ПГПТ может быть причиной выявляемых различий в базальных уровнях и метаболизме витамина D.

Половые стероиды, в частности эстрогены, стимулируют синтез DBP. Это объясняет, почему общие концентрации витамина D выше во время беременности по сравнению с небеременными, в то время как концентрации свободного витамина D остаются одинаковыми в обеих группах женщин [25]. Подтверждением этого является полученная нами разница в концентрации DBP между мужчинами и женщинами.

CYP24A1 экспрессируется в тканях, которые считаются мишенями для витамина D и ПТГ, включая почки, кишечник и кости. Транскрипция гена CYP24A1 заметно индуцируется связыванием VDR 1,25(OH)2D3 с рецептором витамина D [26]. За счет регуляции экспрессии гена CYP24A1, кодирующего фермент 24-гидроксилазу, создается система контроля с отрицательной обратной связью, ограничивающая эффекты 1,25(OH)2D3 и возможности активации 24(OH)D3. Фермент метаболизирует 25(OH)D3 и 1,25(OH)2D3 в неактивные формы 24,25(OH)2D3 и 1,24,25(OH)3D3 [27]. Таким образом, 1,25(OH)2D3 регулирует свой собственный метаболизм, защищая от гиперкальциемии и ограничивая уровни 1,25(OH)2D3 в клетках. 1,25(OH)2D3 и отношение 25(OH)D3/24,25(OH)2D3, отражающее активность 24-гидроксилазы, были статистически значимо выше в группе ПГПТ на всех визитах нашего исследования (р<0,001).

В группе ПГПТ перед приемом колекальциферола выявлена прямая взаимосвязь 1,25(OH)2D3 с альбумин-скорректированным и ионизированным кальцием, а также между отношением 25(OH)D3/24,25(OH)2D3 с иПТГ и магнием. Уровни иПТГ и магния были выше при более низком свободном 25(OH)D, а также при более низких 24,25(OH)2D3. По данным Q. Dai и соавт. [28], уровень магния может быть важен для оптимизации статуса 25(OH)D. Коррекция уровня магния крови значимо влияла на достигаемую концентрацию 25(OH)D3, 25(OH)D2 и 24,25(OH)2D3 в зависимости от исходных концентраций 25(OH)D в сыворотке крови, что было связано с повышенным образованием 24,25(OH)2D3 и более низкими достигаемыми уровнями 25(OH)D3 при наличии адекватных концентраций витамина D в крови (с ~30 до 50 нг/мл), чем при уровнях не более 30 нг/мл.

После приема болюсной дозы колекальциферола на всех визитах в группе ПГПТ уровни 1,25(ОН)2D3 и 25(OH)D3/24,25(OH)2D3 значимо увеличивались, а уровни 25(OH)D3/1,25(OH)2D3 — снижались. Это отражает тот факт, что у пациентов с ПГПТ даже после коррекции уровней витамина D сохраняется особенность метаболизма витамина — повышенная активация в почках под действием 1-α-гидроксилазы и повышенная активность 24-гидроксилазы, и, таким образом, это увеличивает метаболические «траты» витамина D, снижая его уровни в крови.

Фермент 25-гидроксивитамин D3-3-эпимераза, находящийся в эндоплазматическом ретикулуме ряда клеток/тканей, но не в почках, осуществляет инверсию 3β → 3α, образуя метаболит 3-epi-25(OH)D3, из которого в дальнейшем может образовываться 3-epi-1,25(OH)2D3, обладающий пониженной биологической активностью по сравнению с 1,25(OH)2D3 [8] вследствие редуцированной в 35–120 раз аффинности связывания c VDR и, как следствие, с ограниченной способностью стимулировать всасывание кальция в кишечнике и стимуляцией экспрессии 24-гидроксилазы. Концентрация 3-epi-25(OH)D3 в сыворотке крови сильно варьирует — от менее 1% до 25%, в отличие от концентрации 25(OH)D3 со средним показателем порядка 4,75% [29] и более высокими значениями у детей — от 8,7 до 61,1% концентрации 25(OH)D3 [30]. 3-epi-1,25(OH)2D3, по-видимому, обладает большей метаболической стабильностью, чем 1,25(OH)2D3, поэтому, несмотря на более низкую биологическую активность, он может иметь более продолжительные эффекты in vivo, в том числе по подавлению секреции ПТГ культивированными паратиреоидами [31]. На основании вышеизложенного 3-epi-25(OH)D3 является менее активным предшественником, чем 25(OH)D3, и не отражает статус витамина D. В связи с этим эти два метаболита следует измерять отдельно, но современные методики как на основе иммуноферментных и иммунохемилюминесцентных анализов, так и LC-MS/MS имеют ряд ограничений и не всегда способны разделять эти два эпимера [32], что приводит к завышению измеряемого 25(OH)D.

Использованная в настоящем исследовании методика позволила достоверно разделить эти метаболиты. Исходные значения 3-epi-25(OH)D3 между группами статистически не различались и составляли 4% vs 10% в группах ПГПТ и контроля соответственно, что полностью сопоставимо с ранее представленными данными G. Lensmeyer и соавт. [29]. После приема колекальциферола уровень 3-epi-25(OH)D3 повышался в обеих группах, но на визитах 3 и 4 его уровни в крови были значимо выше в группе контроля. Динамика увеличения метаболита 3-epi-25(OH)D3 относительно исходного уровня в группе ПГПТ составила 278–411%, а в группе контроля — 204–321%. Образование 3-epi-25(OH)D3 в ходе метаболизма витамина D является еще одним вариантом дополнительного расхода запасов витамина D, но, судя по полученным нами данным, не является существенным при ПГПТ.

Существует мнение, что 25(OH)D в сыворотке крови — оптимальный биохимический маркер нутритивного статуса витамина D. Пока неясно, станет ли свободный 25(OH)D лучшим маркером, чем общий 25(OH)D, но, по данным консенсуса [33], большинство клеток, за исключением клеток почечных канальцев, подвергается воздействию свободного, а не общего 25(OH)D, и предполагается, что именно свободный 25(OH)D может иметь большее значение для локального производства и действия 1,25(OH)2D, лежащего в основе плейотропных эффектов витамина D. Исходные уровни свободного 25(OH)D не отличались у пациентов с ПГПТ и практически здоровых лиц, но после введения болюсной дозы колекальциферола отмечено их повышение в 1,6–2,3 раза, что было значимо выше в группе контроля на визитах 2 и 3. Свободный 25(OH)D имел сильную прямую корреляцию с общим 25(OH)D (r=0,86; p<0,0000001) и, в отличие от общего, достоверную обратную корреляцию средней силы с иПТГ (r=-0,43; p<0,003), но не с кальцием сыворотки крови, в т.ч. скорректированным на альбумин. Эти данные подтверждают важную роль свободной формы витамина D не только в реализации своих плейотропных эффектов, но и в контроле секреции ПТГ в околощитовидных железах, независимо от повышения кальциемии [34].

В нашей работе также получены данные по фармакокинетике колекальциферола в исследуемые точки времени визитов. В исходной точке в обеих группах наблюдались практически неопределяемые значения колекальциферола (0,01 нг/мл), тогда как на визите 2 его концентрация значимо повышалась до 182,5 и 226 пг/мл с дальнейшим снижением до 21,3 и 23,5 пг/мл в группах ПГПТ и контроля соответственно. Профиль фармакокинетики колекальциферола свидетельствует о наличии быстрой конверсии нативного витамина в его метаболиты, что не приводит к повышению уровня колекальциферола в крови, соответствующему принятой дозе, при этом сохраняется его значимое повышение в крови на 1-е сутки, и меньшее — на 3 и 7-е сутки.

В настоящем исследовании мы получили достоверно значимые отличия повышения уровней кальциемии на последующих визитах, не превышавших 0,05 ммоль/л от исходных значений, что, безусловно, является следствием увеличения всасывания кальция в кишечнике и его реабсорбции в почках, однако не имеет клинической значимости. В группе контроля зафиксировано аналогичное повышение кальция на 0,04 ммоль/л с пиком на визите 2. При этом уровень кальциурии при сравнении визитов в обеих группах значимо не менялся. Вопрос о схемах терапии колекальциферолом у пациентов с ПГПТ пока остается дискутабельным. По результатам метаанализа 10 наблюдательных исследований (n=340), использование данного препарата в насыщающих дозировках (до 100 000 МЕ в неделю) на этапе предоперационной подготовки сопровождалось снижением уровня иПТГ, достоверным увеличением 25(ОН)D и сохранением исходных показателей сывороточного кальция и суточной кальциурии в большинстве случаев. В 2,2% случаев зарегистрировано нарастание гиперкальциемии, повлекшее за собой отмену препаратов. Рандомизированное двойное слепое исследование, посвященное оценке эффективности и безопасности назначения колекальциферола в дозе 2800 МЕ в сутки в течение 6 мес до и после операции, продемонстрировало значимое снижение уровня исходного иПТГ c достижением оптимальных значений 25(ОН)D, при этом показатели кальциемии и суточной кальциурии не изменялись [11].

Клиническая значимость результатов

Впервые проведена комплексная оценка метаболитов витамина D у пациентов с ПГПТ до и после использования болюсной дозы колекальциферола. Полученные результаты, свидетельствующие об особенностях метаболизма витамина D, в условиях хронической избыточной секреции ПТГ имеют высокую значимость для понимания патогенеза заболевания, а также могут быть использованы для разработки терапевтических схем назначения колекальциферола в указанной популяции.

Ограничения исследования

Основными ограничениями исследования выступали малые объемы выборок группы пациентов и группы здорового контроля, что могло сыграть роль в отсутствии четких связей между главными показателями. Также наблюдалось смещение по возрасту группы контроля в сторону более молодых участников.

Направления дальнейших исследований

Определение прогностической ценности свободного 25(OH)D, 25(OH)D3, 24,25(OH)2D3, 3-epi-25(OH)D3 и других маркеров и метаболитов витамина D требует дальнейшего исследования.

## ЗАКЛЮЧЕНИЕ

Информация по метаболитам витамина D и их взаимосвязи с основными показателями фосфорно-кальциевого обмена у пациентов с верифицированным ПГПТ существенно ограничена, что послужило поводом для проведения настоящего исследования. В нашей работе у 69% пациентов с ПГПТ уровень 25(ОН)D соответствовал дефициту витамина D (менее 20 нг/мл). В группе пациентов с ПГПТ перед приемом колекальциферола наблюдалась прямая ассоциация 1,25(OH)2D3 с альбумин-скорректированным и ионизированным кальцием, а также между отношением 25(OH)D3/24,25(OH)2D3 с иПТГ и магнием, что позволяет предположить участие магния в достижении целевых показателей 25(OH)D. В группе ПГПТ уровни 1,25(ОН)2D3 и соотношение 25(OH)D3/24,25(OH)2D3 значимо увеличивались на всех визитах после приема болюсной дозы колекальциферола, а соотношение 25(OH)D3/1,25(OH)2D3 снижалось. Это отражает тот факт, что даже после коррекции уровней витамина D у пациентов с ПГПТ сохраняется особенность метаболизма витамина — его повышенная активация в почках под действием 1-α-гидроксилазы и усиленная активность 24-гидроксилазы. Использование насыщающих доз колекальциферола в группе ПГПТ не приводило к значимому нарастанию гиперкальциемии и гиперкальциурии, что свидетельствует о безопасности использования данной схемы у пациентов с исходно мягкой гиперкальциемией (альбумин-скорректированный кальций <3 ммоль/л). Определение прогностической ценности определения уровней свободного 25(OH)D, 25(OH)D3, 24,25(OH)2D3, 3-epi-25(OH)D3 и других маркеров требует дальнейшего уточнения.

## ДОПОЛНИТЕЛЬНАЯ ИНФОРМАЦИЯ

Источники финансирования. Исследование выполнено при поддержке Российского научного фонда (проект номер 19-15-00243).

Конфликт интересов. Авторы декларируют отсутствие явных и потенциальных конфликтов интересов, связанных с содержанием настоящей статьи.

Участие авторов. Маганева И.С. — разработка концепции исследования, проведение обследования участников исследования, сбор материала, анализ данных, написание текста статьи; Пигарова Е.А. — разработка концепции исследования, проведение обследования участников исследования, сбор материала, анализ данных, написание текста статьи; Шульпекова Н.В. — проведение обследования участников исследования, сбор материала, анализ данных, написание текста статьи; Рожинская Л.Я. — разработка концепции исследования, сбор материала и анализ данных, подготовка статьи к публикации; Дзеранова Л.К. — разработка концепции исследования, проведение обследования участников исследования, сбор материала, подготовка статьи к публикации; Еремкина А.К — анализ данных и литературы, написание текста статьи; Милютина А.П.— обработка материала, статистический анализ данных; Жуков А.Ю. — разработка концепции исследования, проведение обследования участников исследования, сбор материала, подготовка статьи к публикации; Поваляева А.А. — разработка концепции исследования, проведение обследования участников исследования, сбор материала, подготовка статьи к публикации; Богданов В.П. — проведение лабораторного исследования образцов биоматериала, редактирование текста статьи; Мокрышева Н.Г. — разработка концепции исследования, анализ данных, редактирование текста статьи.

Все авторы одобрили финальную версию статьи перед публикацией, выразили согласие нести ответственность за все аспекты публикации, подразумевающую надлежащее изучение и решение вопросов, связанных с точностью или добросовестностью любой части работы.
